# Implant geometry and detection rates of prostate fiducial markers after transrectal ultrasound-guided perineal implantation for image-guided 6D-tracking in robotic stereotactic body radiotherapy

**DOI:** 10.1007/s00066-024-02363-y

**Published:** 2025-02-06

**Authors:** Arne Grün, Katharina Heil, Daniel Zips, Goda Kalinauskaite, Dirk Böhmer

**Affiliations:** 1 Department for Radiation Oncology, Charité—Universitaetsmedizin Berlin, Corporate member of Freie Universitaet Berlin, Humboldt-Universitaet zu Berlin and Berlin Institute of Health, Campus Virchow-Klinikum, Augustenburger Platz 1, 13353 Berlin, Germany; 2 Department for Radiation Oncology, Charité—Universitaetsmedizin Berlin, corporate member of Freie Universitaet Berlin, Humboldt-Universitaet zu Berlin and Berlin Institute of Health, Berlin, Germany; 3 Department for Radiation Oncology, Charité—Universitaetsmedizin Berlin, corporate member of Freie Universitaet Berlin, Humboldt-Universitaet zu Berlin, and Berlin Institute of Health, Campus Benjamin Franklin, Berlin, Germany

**Keywords:** Toxicity, Quality-Assurance, Intra-Prostatic, Gold-Seeds, SABR

## Abstract

**Purpose:**

Fiducial markers (FM) are essential in prostate robotic stereotactic body radiotherapy (SBRT). Accuray® (Madison, WI, USA) provides an implantation guideline for reliable detection. We report on complication rates and analyze how the geometrical implantation quality correlated with subsequent detection rates. We also investigated whether factors such as single vs. double FM, body mass index (BMI), prostatic gland volume, and implantation-to-treatment interval were predictive for geometry and detection quality.

**Methods:**

A retrospective analysis of 64 patients receiving transrectal ultrasound (TRUS)-guided transperineal implantation of ≥ 3 prostate FM and robotic SBRT between January 2011 and May 2021 was performed. Adverse events (AE) were classified according to the Society of Interventional Radiology (SIR) classification system. Digitally reconstructed radiographs (DRR) and the planning CT constituted the basis for implant geometry calculations. Marker detection rates were obtained from the Synchrony® (Accuray®) log.

**Results:**

Complication rates were low, with mostly mild AE. Double FM significantly improved the rate of obtaining an optimal implantation geometry. High FM detection rates during treatment could be achieved independent of implantation geometry and type of FM. BMI and prostatic gland volume did not correlate with geometry and detection quality. An implantation-to-treatment interval of > 42 days was predictive for lower detection rates.

**Conclusion:**

Transperineal intraprostatic FM implantation is a safe procedure. We recommend the use of double markers for reduction of trauma (two punctures instead of four) and, hence, increased patient comfort. Double FM were significantly predictive for achieving an optimal implantation geometry, which was borderline significant for improved marker detection rates over the course of the five-fraction treatment.

**Supplementary Information:**

The online version of this article (10.1007/s00066-024-02363-y) contains supplementary material, which is available to authorized users.

## Introduction

**Fig. 1:**
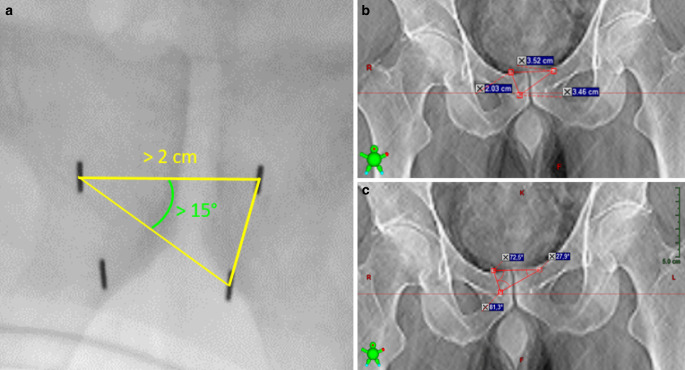
Triangulation over fiducial markers. **a** Connecting the center of mass of at least three fiducials yields a triangle whose side lengths and angles within the triangle shall exceed 2 cm and 15°, respectively, enabling the tracking system of the Cyberknife® (Accuray® Inc., Madison, WI, USA) to safely tell them apart and be able to calculate the uncertainty. Measurement of interfiducial distances (**b**) and angles (**c**) in a digitally reconstructed radiograph obtained from the planning CT

Intraprostatic fiducial markers (FM) have been shown to be a reliable surrogate for prostate gland motion [1] and allow for the reduction of safety margins [2]. This, in turn, has paved the way for ultrahypofractionated prostate stereotactic body radiotherapy (SBRT) [3], which has been shown to be noninferior with respect to failure-free survival compared to conventionally fractionated external beam radiotherapy in low- to high-risk prostate cancer patients [4, 5]. The CyberKnife® system (Accuray® Inc., Madison, WI, USA) is equipped with orthogonal X‑rays for target visualization and real-time tracking through the Synchrony® system [6]. Off-set values between the Synchrony® X‑rays and the digitally reconstructed radiograph (DRR) from the planning computed tomography (CT) can be corrected by the six-dimensional (6D) couch and the robotic arm. At least three FM are necessary to allow for 6D motion tracking. Accuray® recommends implanting four FM for redundancy [7, 8] based on Murphy et al. [9]. An optimal implantation geometry with sufficient space between the FM exceeding the colinearity threshold permits rigid body error (RBE) calculation. In a 2D projection of the markers, the side lengths of a triangle between three markers (the centers of fiducial masses constitute the vertices) and the internal angles in the same triangle should exceed 2 cm and 15°, respectively (Fig. 1). In conjunction with the internal delivery and tracking precision of < 0.5 and 1 mm, respectively, planning target volume (PTV) margins can thus be significantly reduced [10].

We describe the implantation procedure, complication rates, and fraction-to-fraction detection rates during treatment. From our own experience, the system robustly detects prostate fiducials even if the targeted implantation geometry could not be achieved; hence, we analyzed whether implantation geometry and detection rates during treatment correlated. Furthermore, we analyzed whether the use of single fiducials versus connected double fiducials would impact the achievement of an optimal implantation geometry and detection rates. Finally, we looked at factors potentially impacting implantation geometry and detection rates such as body mass index (BMI) [11, 12], prostatic gland volume [13], and the implantation-to-treatment interval.

## Methods

**Fig. 2:**
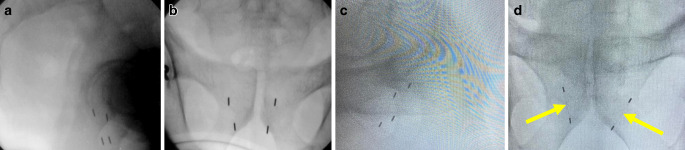
Types of fiducials. **a**, **b** Lateral and anteroposterior (a/p) postimplant X‑rays of four single intraprostatic fiducials; **c**, **d** lateral and a/p postimplant X‑rays of four intraprostatic fiducials, two markers on each side are connected by a thin titanium wire (arrows) keeping the nodes 2 cm apart

A retrospective analysis of 64 patients receiving transrectal ultrasound (TRUS)-guided transperineal implantation of at least three prostate FM and subsequent robotic SBRT for localized prostate cancer between 01.01.2011 and 26.05.2021 at the Charité. All treatments were conducted according to Good Clinical Practice and the Charité Radiation Protection Laws. The research complied with the Declaration of Helsinki. The ethics board of the Charité approved the study (EA1/321/21). Since data were stored anonymously, the informed consent requirement was waived. Prior to the procedure, warfarin-like anticoagulants were switched to heparin and antiplatelet drugs were paused [14]. A rectal enema (Mikroklist® or Microlax®; Johnson & Johnson GmbH OTC, Neuss, Germany, EAN 9088883907556) was prescribed ~2 h prior to the implantation. The ultrasound probe was fixed on a floor-mounted brachytherapy stabilizer and stepper (SurePoint™; Cincinnati, OH, USA, GTIN 00801741079016). The implantation was performed under local anesthesia (lidocaine) and a prophylactic shot of i.v. antibiotics (ampicillin/sulbactam) was given. All implantation procedures during the first year were done by DB. All subsequent implantations were done by AG. The target geometry was according to Holmes et al. [15], who refined the initial Accuray® guideline specifically for prostate robotic SBRT. The aim was to implant two FM peripherally in each prostate lobe with sufficient interfiducial space and in the same coronal plane to exceed the colinearity threshold. We initially used single FM but eventually switched to two pairs of Fleximark® gold-on-titanium fiducials with two nodes and 20 mm spacing preloaded in an 18 gage (GA) by a 20 cm sterile needle (Riverpoint Medical, LLC, Portland, OR, USA) for reduction of invasive trauma, enhancement of patient comfort, and reduction of procedure time (Fig. 2). Postimplant anteroposterior (a/p) and lateral X‑rays were obtained. Potential complications were documented. To account for posttraumatic swelling, the planning CT (Somatom, Siemens Healthineers, Forchheim, Germany) (slice thickness 1 mm, supine position, hands on chest) was obtained approximately 1 week later. An a/p DRR and the planning CT constituted the basis for our implantation geometry calculations. During treatment, Synchrony® creates a log of the exact FM used for tracking which can later be accessed for analysis.

For subgroup analysis, we categorized patients with respect to their BMI (18.5–24.9; 25.0–29.9; 30.0–34.9 kg/m^2^), prostate volume (≤ 29.9; 30.0–59.9; 60.0–89.9; 90.0–119.9; ≥ 120 cc) and implantation-to-treatment time interval (< 15; 15–< 28; 28–< 42; ≥ 42 days). BMI classification is according to the official grading system, the prostate volume groups were devised by starting with a normal volume of up to 30 ml and then proceeding in 30 ml steps, the interval groups were devised by using 2‑week intervals.

Adverse events (AE) were graded according to the Society of Interventional Radiologists (SIR) classification system for complications [16].

Continuous variables are presented as median and range, categorical variables as absolute and relative frequencies. The number of missing values is given. Comparisons between groups were performed either by Fishers exact test (categorical variables) or t‑tests/Welch tests (continuous variables). A *p*-value < 0.05 was considered significant. Due to entries with “0” and/or standard deviations of 0, in some cases, *p*-values could not be calculated. All calculations were done using R version 4.3.1 (R Foundation, Vienna, Austria).

## Results

All 84 patients scheduled to receive prostate FM implantation between 01.01.2011 and 26.05.2021 were initially evaluated for inclusion in this study. Twenty patients were excluded (procedure not performed [*n* = 1], implantation of less than three markers [*n* = 19]). Hence, the study cohort consists of 64 male patients with at least three intraprostatic fiducials receiving robotic SBRT for localized prostate cancer (Table 1). All patients were Eastern Cooperative Oncology Group (ECOG) 0–1. Because of improper marker positioning, two patients needed additional implantation of one fiducial, one patient needed two extra fiducials. Procedure time including documentation is around 20–30 min, depending on complications and the use of single versus double FM. No seeds were lost.

**Table 1:** Patients’ characteristics

Number of patients: 64	
*Age (years)*	
Median (range)	71 (56–81)
Missing	0
*Gleason score*	
6	12 (20.0%)
7a	32 (53.3%)
7b	9 (15.0%)
8	4 (6.7%)
9	3 (5.0%)
Missing	4
*Weight (kg)*	
Median (range)	83.5 (60.0–105.5)
Missing	16
*Height (cm)*	
Median (range)	178.5 (166.0–196.0)
Missing	16
*Body mass index (kg/m*^*2*^*)*	
Median (range)	25.9 (20.8–33.2)
Missing	16
*Implantation-to-treatment interval (days)*	
Median (range)	17.0 (6.0–188.0)
Missing	0
*Primary or recurrent prostate cancer*	
Primary	49 (76.6%)
Recurrent	15 (23.4%)
Missing	0
*Prostate volume (cc)*	
Median (range)	56.6 (10.3–132.4)
Missing	0
*Type of fiducials*	
Double	55 (85.9%)
Single	9 (14.1%)
Missing	0
*Number of fiducials per patient*	
4	60 (93.8%)
3	1 (1.6%)
5	2 (3.1%)
6	1 (1.6%)
Missing	0

The whole cohort consisted of 64 patients with either primary or recurrent prostate cancer undergoing transrectal ultrasound-guided perineal implantation of gold fiducials into the prostate for image-guided stereotactic body radiotherapy

Following the Accuray® (Madison, WI, USA) guideline for marker tracking, the aim was to implant at least three fiducials spaced appropriately apart from one another

Initially we started out using single fiducials but eventually switched to using two-node gold-on-titanium fiducial markers preloaded in a single needle

We recorded only mild AE such as pain and transient hematuria, which subsided without intervention within a day or two after the procedure and had no impact on planning and treatment. One patient developed prostatitis and had to undergo antibiotic treatment (since this could be interpreted as either non-substantial or substantial treatment, we graded it as mild to moderate). All of these adverse events were caused by the procedure. None of the patients had higher-class complications that required hospitalization. None of the procedures had to be interrupted due to complications (Table 2).

**Table 2:** Adverse events

Severity description	Mild	Moderate	Severe	Life-threatening	Death
Pain	23	0	0	0	0
Hematuria	18	0	0	0	0
Prostatitis	1		0	0	0

Description and severity classification are according to the Society of Interventional Radiology classification system for adverse events (AE), part A

Part B classification for all AEs was: A. causality: category 3: AE caused by the procedure B

Patient and procedural risk modifier: category 3: no modifier C

AE preventability: category 1: rarely preventable, i.e., well-described and “typical” for the procedure and occurring despite adequate precautionary and preventive measures D

AE management: category 1: most operators would have handled the AE similarly

Detection rates compared between patients with all or at least one triangle vs. not all or not at least one triangle meeting the optimal geometry criteria did not differ (Table 3 and Online Supplement Table 1), although there is a trend towards a difference between the groups from fractions one to five, with the number of patients in whom all FM could be detected dropping more markedly in the “not all ∆ met criteria” group.

**Table 3:** Fiducial detection rate by patients with all digitally reconstructed radiograph triangles having met the distance/angle criteria vs. patients in whom not all triangles met the criteria. *N* = the whole cohort (64)

	All ∆ met criteria (*n* = 41)	Not all ∆ met criteria (*n* = 23)	Total (*n* = 64)	*p*-value
*Fraction # 1*				
All detected	39 (95.1%)	22 (95.7%)	61 (95.3%)	0.92
1 fiducial not detected	2 (4.9%)	1 (4.3%)	3 (4.7%)	
2 fiducials not detected	0 (0.0%)	0 (0.0%)	0 (0.0%)	
3 fiducials not detected	0 (0.0%)	0 (0.0%)	0 (0.0%)	
4 fiducials not detected	0 (0.0%)	0 (0.0%)	0 (0.0%)	
Missing	0	0	0	
*Fraction # 2*				
All detected	38 (92.7%)	20 (87.0%)	58 (90.6%)	0.15
1 fiducial not detected	3 (7.3%)	1 (4.3%)	4 (6.2%)	
2 fiducials not detected	0 (0.0%)	2 (8.7%)	2 (3.1%)	
3 fiducials not detected	0 (0.0%)	0 (0.0%)	0 (0.0%)	
4 fiducials not detected	0 (0.0%)	0 (0.0%)	0 (0.0%)	
Missing	0	0	0	
*Fraction # 3*				
All detected	39 (95.1%)	18 (78.3%)	57 (89.1%)	0.07
1 fiducial not detected	2 (4.9%)	3 (13.0%)	5 (7.8%)	
2 fiducials not detected	0 (0.0%)	2 (8.7%)	2 (3.1%)	
3 fiducials not detected	0 (0.0%)	0 (0.0%)	0 (0.0%)	
4 fiducials not detected	0 (0.0%)	0 (0.0%)	0 (0.0%)	
Missing	0	0	0	
*Fraction # 4*				
All detected	37 (92.5%)	17 (73.9%)	54 (85.7%)	0.07
1 fiducial not detected	3 (7.5%)	4 (17.4%)	7 (11.1%)	
2 fiducials not detected	0 (0.0%)	2 (8.7%)	2 (3.2%)	
3 fiducials not detected	0 (0.0%)	0 (0.0%)	0 (0.0%)	
4 fiducials not detected	0 (0.0%)	0 (0.0%)	0 (0.0%)	
Missing	1	0	1	
*Fraction # 5*				
All detected	35 (92.1%)	16 (72.7%)	51 (85.0%)	0.07
1 fiducial not detected	3 (7.9%)	4 (18.2%)	7 (11.7%)	
2 fiducials not detected	0 (0.0%)	2 (9.1%)	2 (3.3%)	
3 fiducials not detected	0 (0.0%)	0 (0.0%)	0 (0.0%)	
4 fiducials not detected	0 (0.0%)	0 (0.0%)	0 (0.0%)	
Missing	3	1	4	

*N* = the whole cohort (64)

There was no statistical difference between the groups over the course of the five-fraction treatment, although there is a trend in difference between the groups from fraction one to five with the number of patients in whom all markers could be detected, dropping more markedly in the “not all ∆ met criteria” group

The rate in which patients with double versus single FM had all or at least one triangle meeting the proposed criteria was significantly different in favor of the connected double FM. No patients with single FM had a complete set of correct triangles and only 33.3% hat at least one correct triangle (Table 4).

**Table 4:** All vs. not all digitally reconstructed radiograph triangles meeting the distance/angle criteria in dependence of using double or single markers

	Double (*n* = 55)	Single (*n* = 9)	Total (*n* = 64)	*p*-value
*All ∆ met criteria*				
Yes	41 (74.5%)	0 (0.0%)	41 (64.1%)	< 0.01
No	14 (25.5%)	9 (100.0%)	23 (35.9%)	
Missing	0	0	0	
*At least one ∆ met criteria*				
Yes	53 (96.4%)	3 (33.3%)	56 (87.5%)	< 0.01
No	2 (3.6%)	6 (66.7%)	8 (12.5%)	
Missing	0	0	0	

At least one vs. not at least one hypothetical digitally reconstructed radiograph (DDR) triangle meeting the distance/angle criteria in dependence of using double or single markers

Double markers were statistically superior in terms of yielding only or at least one triangle in the DRR that met the distance/angle criteria

Neither BMI nor prostatic gland volume groups had an impact on all or at least one triangle meeting the optimal geometry criteria (Online Supplement Tables 2 and 3), although there seemed to be a negative impact on detectability in patients with very small and very large glands.

The fiducial detection rate over the course of the five-fraction treatment was not different between patients with double or single FM (Table 5), but the rate of detecting all FM drops towards the end of the treatment, with a corresponding increase in the number of patients with one or two FM undetected.

**Table 5:** Detection rate and number of markers detected over the course of the five-fraction treatment in dependance of double or single markers

	Double (*n* = 55)	Single (*n* = 9)	Total (*n* = 64)	*p*-value
*Fraction # 1*				
All detected	53 (96.4%)	8 (88.9%)	61 (95.3%)	0.33
1 fiducial not detected	2 (3.6%)	1 (11.1%)	3 (4.7%)	
2 fiducials not detected	0 (0.0%)	0 (0.0%)	0 (0.0%)	
3 fiducials not detected	0 (0.0%)	0 (0.0%)	0 (0.0%)	
4 fiducials not detected	0 (0.0%)	0 (0.0%)	0 (0.0%)	
Missing	0	0	0	
*Fraction # 2*				
All detected	50 (90.9%)	8 (88.9%)	58 (90.6%)	0.70
1 fiducial not detected	3 (5.5%)	1 (11.1%)	4 (6.2%)	
2 fiducials not detected	2 (3.6%)	0 (0.0%)	2 (3.1%)	
3 fiducials not detected	0 (0.0%)	0 (0.0%)	0 (0.0%)	
4 fiducials not detected	0 (0.0%)	0 (0.0%)	0 (0.0%)	
Missing	0	0	0	
*Fraction # 3*				
All detected	49 (89.1%)	8 (88.9%)	57 (89.1%)	0.79
1 fiducial not detected	4 (7.3%)	1 (11.1%)	5 (7.8%)	
2 fiducials not detected	2 (3.6%)	0 (0.0%)	2 (3.1%)	
3 fiducials not detected	0 (0.0%)	0 (0.0%)	0 (0.0%)	
4 fiducials not detected	0 (0.0%)	0 (0.0%)	0 (0.0%)	
Missing	0	0	0	
*Fraction # 4*				
All detected	46 (85.2%)	8 (88.9%)	54 (85.7%)	0.84
1 fiducial not detected	6 (11.1%)	1 (11.1%)	7 (11.1%)	
2 fiducials not detected	2 (3.7%)	0 (0.0%)	2 (3.2%)	
3 fiducials not detected	0 (0.0%)	0 (0.0%)	0 (0.0%)	
4 fiducials not detected	0 (0.0%)	0 (0.0%)	0 (0.0%)	
Missing	1	0	1	
*Fraction # 5*				
All detected	45 (86.5%)	6 (75.0%)	51 (85.0%)	0.40
1 fiducial not detected	5 (9.6%)	2 (25.0%)	7 (11.7%)	
2 fiducials not detected	2 (3.8%)	0 (0.0%)	2 (3.3%)	
3 fiducials not detected	0 (0.0%)	0 (0.0%)	0 (0.0%)	
4 fiducials not detected	0 (0.0%)	0 (0.0%)	0 (0.0%)	
Missing	3	1	4	

*N* = the whole cohort (64)

There was no statistical difference between the two groups

The rate of detecting all markers drops towards the end of the treatment, with a corresponding increase in the number of patients with one or two markers undetected

Neither BMI groups nor prostate volume groups nor the interval from implantation to treatment groups were predictive for the detection rate over the course of the five-fraction treatment (Table 6 and Online Supplement Tables 4 and 5). There was a significant difference in the rate of 100% detected FM favoring a shorter interval between implantation and treatment (< 15 d, ≥ 15–27 d) with 84.6% and 85.7%, respectively, versus 50% at an interval of ≥ 42 days.

**Table 6:** Fiducial detection rate by interval from implantation to start of treatment groups

	Interval < 15 d (*n* = 13)	15 d < = interval < 28 d (*n* = 42)	28 d < = interval < 42 d (*n* = 7)	Interval > = 42 d (*n* = 2)	Total (*n* = 64)	*p*-value
*Fraction # 1*						
All detected	13 (100.0%)	40 (95.2%)	6 (85.7%)	2 (100.0%)	61 (95.3%)	–
1 fiducial not detected	0 (0.0%)	2 (4.8%)	1 (14.3%)	0 (0.0%)	3 (4.7%)	
2 fiducials not detected	0 (0.0%)	0 (0.0%)	0 (0.0%)	0 (0.0%)	0 (0.0%)	
3 fiducials not detected	0 (0.0%)	0 (0.0%)	0 (0.0%)	0 (0.0%)	0 (0.0%)	
4 fiducials not detected	0 (0.0%)	0 (0.0%)	0 (0.0%)	0 (0.0%)	0 (0.0%)	
Missing	0	0	0	0	0	
*Fraction # 2*						
All detected	13 (100.0%)	37 (88.1%)	6 (85.7%)	2 (100.0%)	58 (90.6%)	–
1 fiducial not detected	0 (0.0%)	3 (7.1%)	1 (14.3%)	0 (0.0%)	4 (6.2%)	
2 fiducials not detected	0 (0.0%)	2 (4.8%)	0 (0.0%)	0 (0.0%)	2 (3.1%)	
3 fiducials not detected	0 (0.0%)	0 (0.0%)	0 (0.0%)	0 (0.0%)	0 (0.0%)	
4 fiducials not detected	0 (0.0%)	0 (0.0%)	0 (0.0%)	0 (0.0%)	0 (0.0%)	
Missing	0	0	0	0	0	
*Fraction # 3*						
All detected	13 (100.0%)	37 (88.1%)	5 (71.4%)	2 (100.0%)	57 (89.1%)	–
1 fiducial not detected	0 (0.0%)	3 (7.1%)	2 (28.6%)	0 (0.0%)	5 (7.8%)	
2 fiducials not detected	0 (0.0%)	2 (4.8%)	0 (0.0%)	0 (0.0%)	2 (3.1%)	
3 fiducials not detected	0 (0.0%)	0 (0.0%)	0 (0.0%)	0 (0.0%)	0 (0.0%)	
4 fiducials not detected	0 (0.0%)	0 (0.0%)	0 (0.0%)	0 (0.0%)	0 (0.0%)	
Missing	0	0	0	0	0	
*Fraction # 4*						
All detected	11 (84.6%)	37 (88.1%)	4 (66.7%)	2 (100.0%)	54 (85.7%)	–
1 fiducial not detected	2 (15.4%)	3 (7.1%)	2 (33.3%)	0 (0.0%)	7 (11.1%)	
2 fiducials not detected	0 (0.0%)	2 (4.8%)	0 (0.0%)	0 (0.0%)	2 (3.2%)	
3 fiducials not detected	0 (0.0%)	0 (0.0%)	0 (0.0%)	0 (0.0%)	0 (0.0%)	
4 fiducials not detected	0 (0.0%)	0 (0.0%)	0 (0.0%)	0 (0.0%)	0 (0.0%)	
Missing	0	0	1	0	1	
*Fraction # 5*						
All detected	11 (84.6%)	34 (87.2%)	5 (83.3%)	1 (50.0%)	51 (85.0%)	–
1 fiducial not detected	2 (15.4%)	3 (7.7%)	1 (16.7%)	1 (50.0%)	7 (11.7%)	
2 fiducials not detected	0 (0.0%)	2 (5.1%)	0 (0.0%)	0 (0.0%)	2 (3.3%)	
3 fiducials not detected	0 (0.0%)	0 (0.0%)	0 (0.0%)	0 (0.0%)	0 (0.0%)	
4 fiducials not detected	0 (0.0%)	0 (0.0%)	0 (0.0%)	0 (0.0%)	0 (0.0%)	
Missing	0	3	1	0	4	
*Total undetected fiducials*						
0	11 (84.6%)	36 (85.7%)	5 (71.4%)	1 (50.0%)	53 (82.8%)	0.04
1	0 (0.0%)	1 (2.4%)	0 (0.0%)	1 (50.0%)	2 (3.1%)	
2	2 (15.4%)	0 (0.0%)	1 (14.3%)	0 (0.0%)	3 (4.7%)	
3	0 (0.0%)	1 (2.4%)	0 (0.0%)	0 (0.0%)	1 (1.6%)	
5	0 (0.0%)	2 (4.8%)	1 (14.3%)	0 (0.0%)	3 (4.7%)	
8	0 (0.0%)	2 (4.8%)	0 (0.0%)	0 (0.0%)	2 (3.1%)	
Missing	0	0	0	0	0	
*Total undetected fiducials per group*						
All detected	11 (84.6%)	36 (85.7%)	5 (71.4%)	1 (50.0%)	53 (82.8%)	0.49
At least 1 undetected fiducial	2 (15.4%)	6 (14.3%)	2 (28.6%)	1 (50.0%)	11 (17.2%)	
Missing	0	0	0	0	0	

The interval groups were devised by using two-week intervals

*N* = the whole cohort (64)

There was no significant difference between the groups, nor could a trend be detected over the course of the five-fraction treatment

Overall, the shortest distance and lowest angle between two FM were 0.7 cm and 6.7°, respectively.

## Discussion

We retrospectively analyzed 64 men with prostate cancer undergoing TRUS-guided transperineal implantation of at least three gold FM into the prostate for image-guided robotic SBRT. Due to the stable team performing the procedure, bias resulting from differing interindividual approaches could be minimized. The procedure is fast, and complication rates were low, with only mild to moderate AE like transient hematuria. Only one patient had to have treatment for an AE (antibiotics for prostatitis). No procedure had to be aborted. No patient had to be hospitalized. This is in line with Kim et al., who reported a major complication rate of 1.3% and a minor complication rate of 20.8% in a cohort of CT- and TRUS-guided implantations. With TRUS-based implantation, they did not see any major complications [17]. A major complication rate at 0–2% is comparable to that of percutaneous biopsies [18] and lower than the rate of CT-guided fiducial implantations, which is up to 5% [19]. In 209 patients with TRUS-guided implantation of four prostate markers, Langenhuijsen reported pain and fever that resolved with oral medication in 6.2%, hematuria lasting more than 3 days in 3.8%, hematospermia in 18.5%, and rectal bleeding in 9.1% [20].

In the initial cohort of 84 patients there were 2 patients (2.4%) who were excluded from this analysis with either misplacement or migration of the FM to outside the prostate. In the 64 patients included in this study, we did not witness any further seed migration. Fawaz et al. reported 0.32% seed migration (2 out of 626) [21].

To our knowledge, this is the first study to analyze factors influencing implantation geometry and subsequent fraction-to-fraction detection rates before and during 6D tracking in robotic prostate SBRT. Cordoba et al. reported on their experience in a cohort of 208 patients (of whom 94, 68, and 36 patients received either an SBRT boost after EBRT, had definitive prostate SBRT, or underwent re-RT after EBRT, respectively) but they focused on complication rates and merely stated that the FM were used for prostate tracking before and during treatment [22]. O’Neill et al. published a lengthy literature review on FM-based prostate IGRT [23]. Although the review cited 50 studies, neither Cordoba et al. nor O’Neill et al. provided any specifics on implant geometry before and during prostate SBRT. Our implantation procedure was based on Holmes et al. [13], who basically provided the actual guideline on prostate fiducial marker implantation by adapting the Accuray® protocol specifically for prostate robotic SBRT. Starting where they left off, we investigated factors influencing the quality of implantation geometry such as the use of single vs. double markers, the interval between implantation and treatment, BMI of the patients, and prostatic gland volume. Furthermore, we provide a fraction-to-fraction analysis of implantation geometry and detection rates. We could show that the procedure itself is safe and devoid of relevant toxicity. When implanting at least three fiducials, the full range of 6D motion tracking can be provided in the majority of cases. This remains true even when the optimal implantation geometry cannot be obtained. The rate at which the optimal implant geometry could be achieved in all or at least one triangle was significantly higher for double FM compared to single FM (74.5 vs. 0% and 96.4 vs. 33.3%, respectively), although the absolute (low) number of single markers might have skewed the results. However, even though no patient with single FM fulfilled the geometry criteria in every triangle, the FM detection rates still remained not significantly different from the patients with double FM. Still, the implant geometry should not be ignored, for patients with a complete set of possible triangles fulfilling the geometry criteria showed a stable detection rate of all FM from fraction one to five of 95.1% to 92.15%, whereas it dropped from 95.7% to 72.7% in patients with suboptimal implant geometry. The rate at which the optimal implant geometry could be achieved was highest in patients with a BMI of 18.5–29.9 kg/m^2^ compared to a BMI of ≥ 30 and in patients with prostatic gland volumes of 30–89.9 cc compared to patients with smaller or larger glands. Furthermore, there was a significant drop in the rate of detecting every FM when the interval from implantation to treatment exceeded 42 days. This might be due to late migration of the seeds or further BPH (benign prostate hyperplasia) growth. With a focus on intrafractional prostate motion, Rose et al. found no significant correlation with age, weight, height, and BMI or rectal, bladder, or prostate volumes [24].

Other forms of FM and image guidance have been explored. Downsides of the Calypso® (Varian Medical Systems, A Siemens Healthineers Company, Palo Alto, Ca, USA) radiofrequency beacon system are the limited accuracy of only 1 mm and the limited range, which is especially problematic in obese patients. Also, due to the size of the beacons, large needles are needed [25]. While noninvasive per se, the RayPilot® (Micropos Medical (publ), Gothenburg, Sweden) system depends on the insertion of a bladder catheter, which reduces patient comfort. While some groups have found the tracking accuracy comparable to that of prostate gold fiducials [26], others have found the accuracy of the system to be inferior to that of the Calypso® system, thus necessitating other imaging modalities for enhanced precision [27]. Vavassori et al. describe a case in which four gold fiducials were implanted via a transperineal approach using CT and electromagnetic navigation (EMN) guidance, which yields high placement accuracy even in out of plane trajectories through virtual navigation [28].

Manual coregistration of prostate FM in the planning CT and planning MRI can yield erroneous offsets of up to 3 mm [29]. In aiming to eliminate such intermodality planning errors, the MIRAGE trial has shown that MRI-guided SBRT is feasible and safe and might even be advantageous with respect to 90-day acute GU and GI toxicity [30], and there are current trials evaluating whether these potential advantages in toxicity allow for further acceleration in ultrahypofractionated prostate SBRT from 5 to 2 fractions [31]. But replacing the orthogonal X‑ray system with MRI guidance seems unpractical in robotic SBRT. Due to the multiple-angle-scanning technique in CyberKnife® SBRT [32], putting the patient in a bore would physically block many of the vantage points necessary for providing highly conformal plans, so the treatment would have to be interrupted and the patient moved. Treatment times per fraction in robotic prostate SBRT range from 30 to > 60 min. The accuracy does not falter towards the end of the session because of the system’s ability to provide repeated X‑ray image guidance for continuous tracking during treatment without long treatment interruptions. This would not be possible with long interruptions, due to protracted image acquisition times.

## Limitations

There are critical issues and shortcomings concerning this analysis. The retrospective nature of this single-center study is susceptible to fault. The cohort size of 64 patients does not preclude selection bias. Also, the group with single FM was underpowered to reliably detect differences to the patients receiving double FM. Also, this was not an exploratory phantom study on how much the proposed geometry criteria could be undercut; hence, exact minimal values cannot be provided.

## Conclusion

Transrectal ultrasound-guided transperineal marker implantation into the prostate is fast and safe, with a low toxicity profile. When implanting at least three fiducials, the detection rates are high even when the optimal implantation geometry cannot not be obtained. We recommend using double FM because the absolute rates of optimal implantation geometries and detection rates are higher. Furthermore, double FM reduce trauma and, hence, increase patient comfort. The implantation procedure time is also shorter. Prostatic gland volume and patient BMI do not negatively correlate with geometry and detection quality. The time from implantation to treatment should not exceed 42 days.

## Supplementary Information

**Table 1. Fiducial detection rate by patients with at least one DRR triangle having met the distance/angle criteria vs. patients with none.** *N* = the whole cohort (64). There was no statistical difference between the groups over the course of the five-fraction treatment, although there was a trend towards a difference between the groups from fraction one to five, with the number of patients in whom all markers could be detected dropping more markedly in the “not all criteria met” group.

**Table 2. All DRR triangles vs. not all DRR triangles detected in dependance of BMI and prostate volume groups.** *N* = the whole cohort (64). The BMI groups were devised according to the official BMI classification. The volume groups were devised by starting with a normal volume of up to 30 ml and then proceeding in 30 ml steps. There was no significant difference between the groups, although there seemed to be a negative impact on detectability in patients with very small and very large glands.

**Table 3. At least one DRR triangle vs. no DRR triangle having met the distance/angle criteria in dependance of BMI and prostate volume groups.** *N* = the whole cohort (64). The BMI groups were devised according to the official BMI classification. The volume groups were devised by starting with a normal volume of up to 30 ml and then proceeding in 30 ml steps. There was no significant difference between the groups, although there seemed to be a negative impact on detectability in patients with very small and very large glands.

**Table 4. Fiducial detection rate by BMI group.** The BMI groups were devised according to the official BMI classification. Only 48 of 64 patients were analyzed due to missing height and/or weight information in 16 patients (see patients’ characteristics). There was no significant difference between the groups, nor could a trend be detected over the course of the five-fraction treatment.

**Table 5. Fiducial detection rate by prostate volume group.** The prostate volume groups were devised by starting with a normal volume of up to 30 ml and then proceeding in 30 ml steps. *N* = the whole cohort (64). There was no significant difference between the groups over the course of the five-fraction treatment, although the overall rate of undetected fiducials increased significantly from fraction one to five.

## Data Availability

All data supporting the results reported in this article are available on a secured data server owned by the Charité—Universitaetsmedizin Berlin, Germany. The datasets used and analyses of all data of this manuscript are available from the corresponding author upon reasonable request.
