# Supplementary material for: Implant geometry and detection rates of prostate fiducial markers after transrectal ultrasound-guided perineal implantation for image-guided 6D-tracking in robotic stereotactic body radiotherapy
Source: Strahlenther Onkol. 2025 Feb 6;201(8):818–27. doi: 10.1007/s00066-024-02363-y (PMC12283462; doi:10.1007/s00066-024-02363-y)
Supplement: Supplementary file 1 — Table 1. Fiducial detection rate by patients with at least one DRR triangle having met the distance/angle criteria vs. patients with none. N = the whole cohort (64). There was no statistical difference between the groups over the course of the five-fraction treatment, although there was a trend towards a difference between the groups from fraction one to five, with the number of patients in whom all markers could be detected dropping more markedly in the “not all criteria met” group. [file 66_2024_2363_MOESM1_ESM.pptx]

## Slide 1
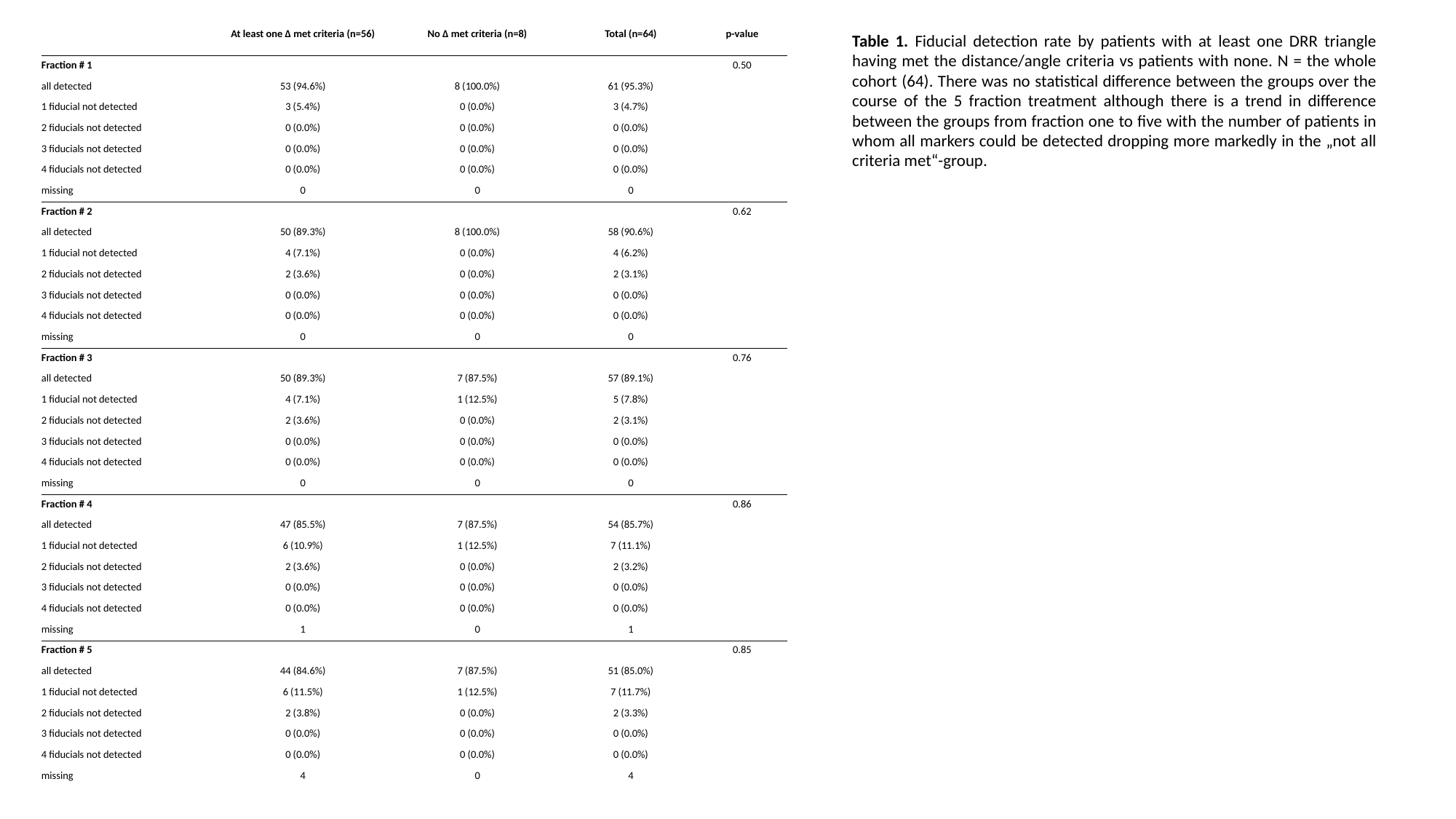

| | At least one Δ met criteria (n=56) | No Δ met criteria (n=8) | Total (n=64) | p-value |
| --- | --- | --- | --- | --- |
| Fraction # 1 | | | | 0.50 |
| all detected | 53 (94.6%) | 8 (100.0%) | 61 (95.3%) | |
| 1 fiducial not detected | 3 (5.4%) | 0 (0.0%) | 3 (4.7%) | |
| 2 fiducials not detected | 0 (0.0%) | 0 (0.0%) | 0 (0.0%) | |
| 3 fiducials not detected | 0 (0.0%) | 0 (0.0%) | 0 (0.0%) | |
| 4 fiducials not detected | 0 (0.0%) | 0 (0.0%) | 0 (0.0%) | |
| missing | 0 | 0 | 0 | |
| Fraction # 2 | | | | 0.62 |
| all detected | 50 (89.3%) | 8 (100.0%) | 58 (90.6%) | |
| 1 fiducial not detected | 4 (7.1%) | 0 (0.0%) | 4 (6.2%) | |
| 2 fiducials not detected | 2 (3.6%) | 0 (0.0%) | 2 (3.1%) | |
| 3 fiducials not detected | 0 (0.0%) | 0 (0.0%) | 0 (0.0%) | |
| 4 fiducials not detected | 0 (0.0%) | 0 (0.0%) | 0 (0.0%) | |
| missing | 0 | 0 | 0 | |
| Fraction # 3 | | | | 0.76 |
| all detected | 50 (89.3%) | 7 (87.5%) | 57 (89.1%) | |
| 1 fiducial not detected | 4 (7.1%) | 1 (12.5%) | 5 (7.8%) | |
| 2 fiducials not detected | 2 (3.6%) | 0 (0.0%) | 2 (3.1%) | |
| 3 fiducials not detected | 0 (0.0%) | 0 (0.0%) | 0 (0.0%) | |
| 4 fiducials not detected | 0 (0.0%) | 0 (0.0%) | 0 (0.0%) | |
| missing | 0 | 0 | 0 | |
| Fraction # 4 | | | | 0.86 |
| all detected | 47 (85.5%) | 7 (87.5%) | 54 (85.7%) | |
| 1 fiducial not detected | 6 (10.9%) | 1 (12.5%) | 7 (11.1%) | |
| 2 fiducials not detected | 2 (3.6%) | 0 (0.0%) | 2 (3.2%) | |
| 3 fiducials not detected | 0 (0.0%) | 0 (0.0%) | 0 (0.0%) | |
| 4 fiducials not detected | 0 (0.0%) | 0 (0.0%) | 0 (0.0%) | |
| missing | 1 | 0 | 1 | |
| Fraction # 5 | | | | 0.85 |
| all detected | 44 (84.6%) | 7 (87.5%) | 51 (85.0%) | |
| 1 fiducial not detected | 6 (11.5%) | 1 (12.5%) | 7 (11.7%) | |
| 2 fiducials not detected | 2 (3.8%) | 0 (0.0%) | 2 (3.3%) | |
| 3 fiducials not detected | 0 (0.0%) | 0 (0.0%) | 0 (0.0%) | |
| 4 fiducials not detected | 0 (0.0%) | 0 (0.0%) | 0 (0.0%) | |
| missing | 4 | 0 | 4 | |
Table 1. Fiducial detection rate by patients with at least one DRR triangle having met the distance/angle criteria vs patients with none. N = the whole cohort (64). There was no statistical difference between the groups over the course of the 5 fraction treatment although there is a trend in difference between the groups from fraction one to five with the number of patients in whom all markers could be detected dropping more markedly in the „not all criteria met“-group.

## Slide 2
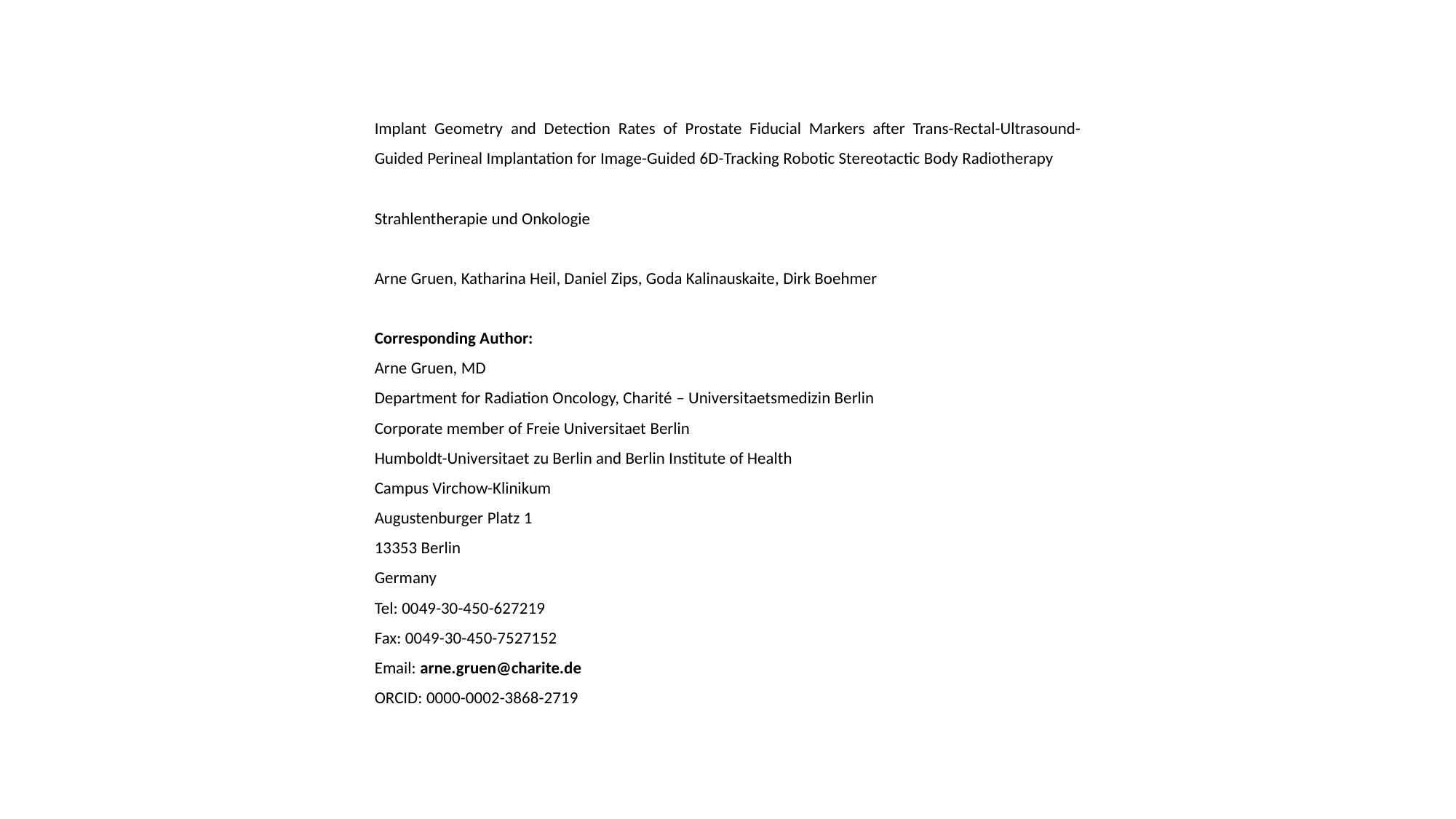

Implant Geometry and Detection Rates of Prostate Fiducial Markers after Trans-Rectal-Ultrasound-Guided Perineal Implantation for Image-Guided 6D-Tracking Robotic Stereotactic Body Radiotherapy
Strahlentherapie und Onkologie
Arne Gruen, Katharina Heil, Daniel Zips, Goda Kalinauskaite, Dirk Boehmer
Corresponding Author:
Arne Gruen, MD
Department for Radiation Oncology, Charité – Universitaetsmedizin Berlin
Corporate member of Freie Universitaet Berlin
Humboldt-Universitaet zu Berlin and Berlin Institute of Health
Campus Virchow-Klinikum
Augustenburger Platz 1
13353 Berlin
Germany
Tel: 0049-30-450-627219
Fax: 0049-30-450-7527152
Email: arne.gruen@charite.de
ORCID: 0000-0002-3868-2719
