# Supplementary material for: Implant geometry and detection rates of prostate fiducial markers after transrectal ultrasound-guided perineal implantation for image-guided 6D-tracking in robotic stereotactic body radiotherapy
Source: Strahlenther Onkol. 2025 Feb 6;201(8):818–27. doi: 10.1007/s00066-024-02363-y (PMC12283462; doi:10.1007/s00066-024-02363-y)
Supplement: Supplementary file 2 — Table 2. All DRR triangles vs. not all DRR triangles detected in dependance of BMI and prostate volume groups. N = the whole cohort (64). The BMI groups were devised according to the official BMI classification. The volume groups were devised by starting with a normal volume of up to 30 ml and then proceeding in 30 ml steps. There was no significant difference between the groups, although there seemed to be a negative impact on detectability in patients with very small and very large glands. [file 66_2024_2363_MOESM2_ESM.pptx]

## Slide 1
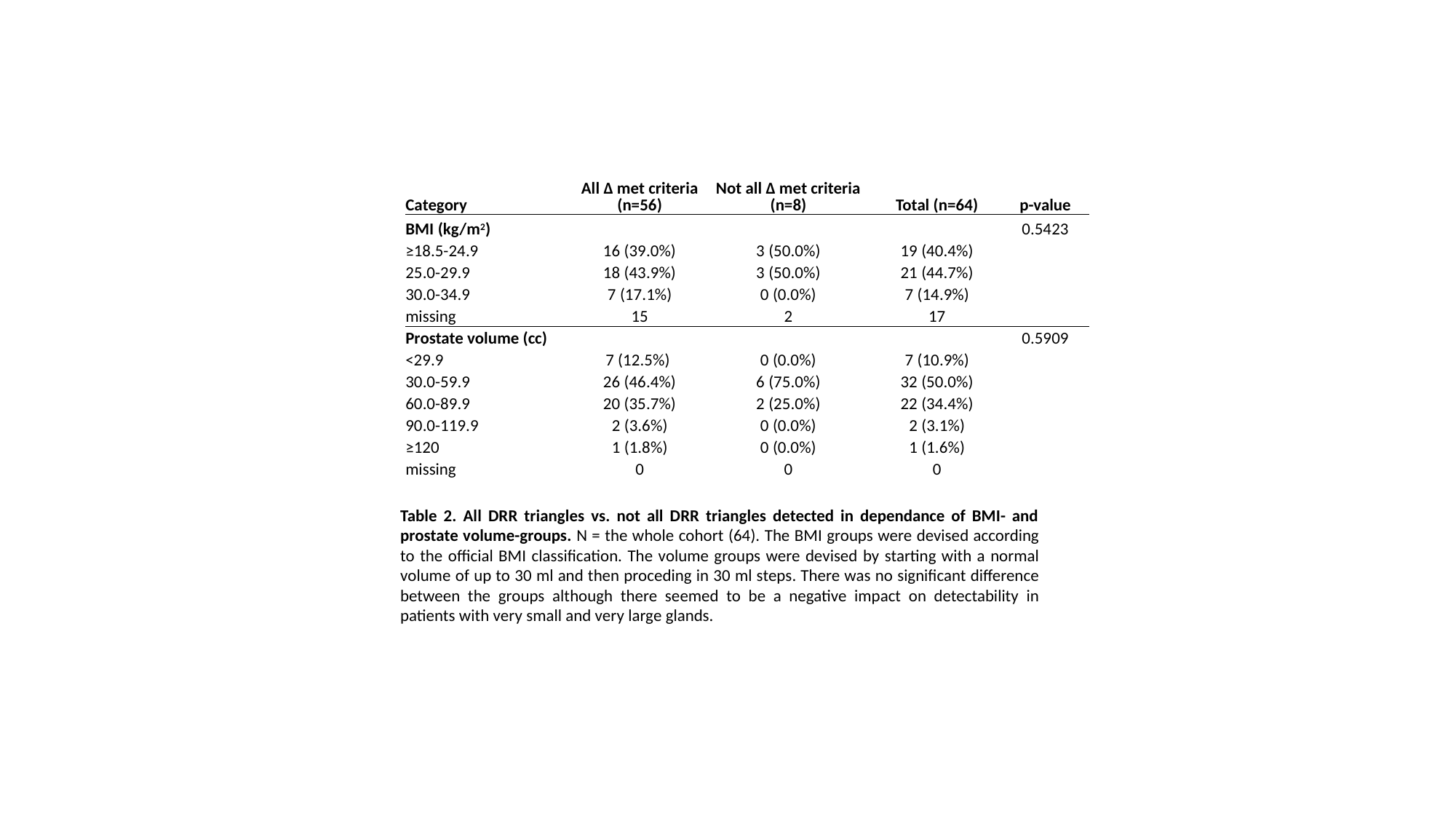

| Category | All Δ met criteria (n=56) | Not all Δ met criteria (n=8) | Total (n=64) | p-value |
| --- | --- | --- | --- | --- |
| BMI (kg/m2) | | | | 0.5423 |
| ≥18.5-24.9 | 16 (39.0%) | 3 (50.0%) | 19 (40.4%) | |
| 25.0-29.9 | 18 (43.9%) | 3 (50.0%) | 21 (44.7%) | |
| 30.0-34.9 | 7 (17.1%) | 0 (0.0%) | 7 (14.9%) | |
| missing | 15 | 2 | 17 | |
| Prostate volume (cc) | | | | 0.5909 |
| <29.9 | 7 (12.5%) | 0 (0.0%) | 7 (10.9%) | |
| 30.0-59.9 | 26 (46.4%) | 6 (75.0%) | 32 (50.0%) | |
| 60.0-89.9 | 20 (35.7%) | 2 (25.0%) | 22 (34.4%) | |
| 90.0-119.9 | 2 (3.6%) | 0 (0.0%) | 2 (3.1%) | |
| ≥120 | 1 (1.8%) | 0 (0.0%) | 1 (1.6%) | |
| missing | 0 | 0 | 0 | |
Table 2. All DRR triangles vs. not all DRR triangles detected in dependance of BMI- and prostate volume-groups. N = the whole cohort (64). The BMI groups were devised according to the official BMI classification. The volume groups were devised by starting with a normal volume of up to 30 ml and then proceding in 30 ml steps. There was no significant difference between the groups although there seemed to be a negative impact on detectability in patients with very small and very large glands.

## Slide 2
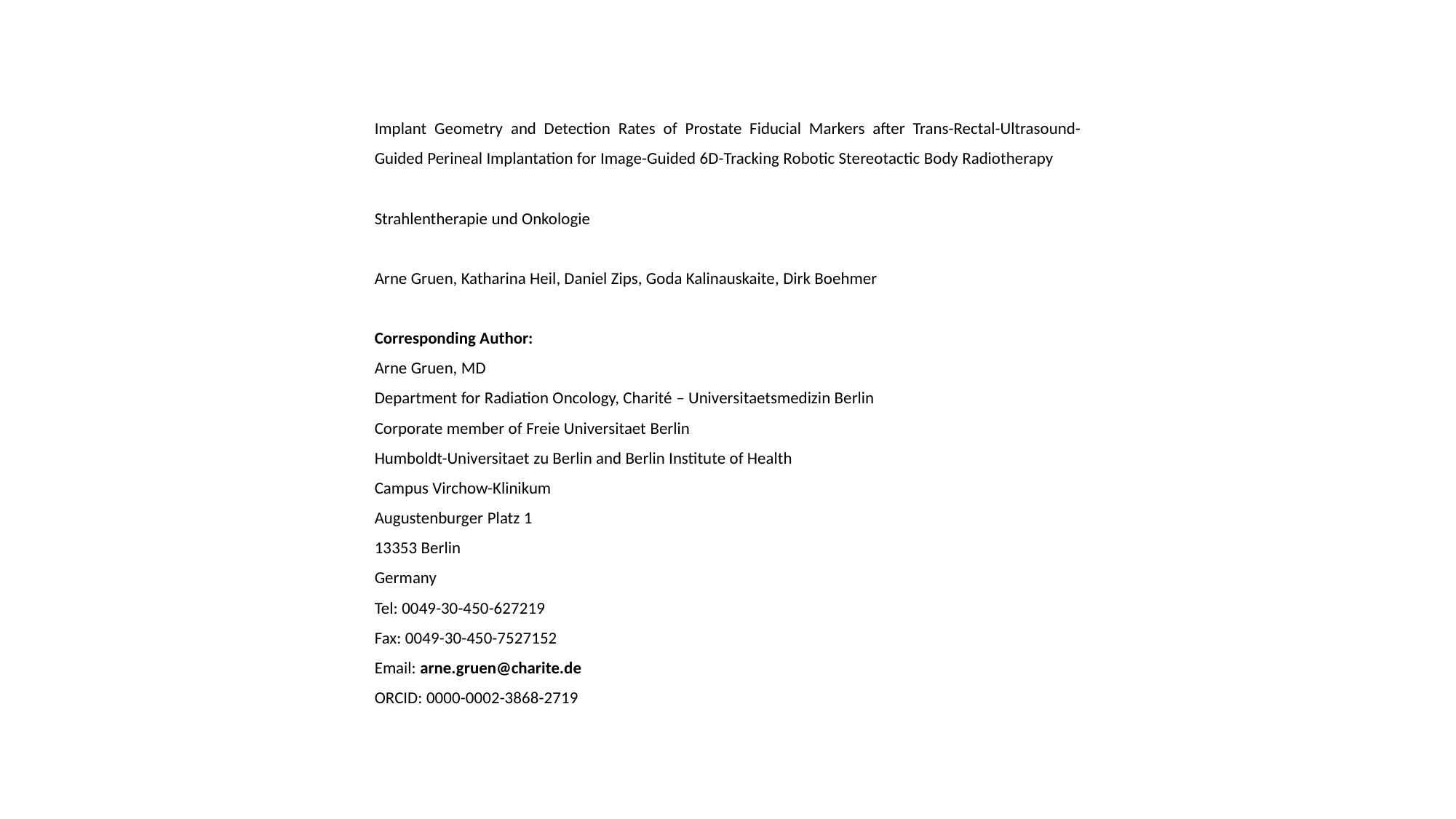

Implant Geometry and Detection Rates of Prostate Fiducial Markers after Trans-Rectal-Ultrasound-Guided Perineal Implantation for Image-Guided 6D-Tracking Robotic Stereotactic Body Radiotherapy
Strahlentherapie und Onkologie
Arne Gruen, Katharina Heil, Daniel Zips, Goda Kalinauskaite, Dirk Boehmer
Corresponding Author:
Arne Gruen, MD
Department for Radiation Oncology, Charité – Universitaetsmedizin Berlin
Corporate member of Freie Universitaet Berlin
Humboldt-Universitaet zu Berlin and Berlin Institute of Health
Campus Virchow-Klinikum
Augustenburger Platz 1
13353 Berlin
Germany
Tel: 0049-30-450-627219
Fax: 0049-30-450-7527152
Email: arne.gruen@charite.de
ORCID: 0000-0002-3868-2719
