# Supplementary material for: Implant geometry and detection rates of prostate fiducial markers after transrectal ultrasound-guided perineal implantation for image-guided 6D-tracking in robotic stereotactic body radiotherapy
Source: Strahlenther Onkol. 2025 Feb 6;201(8):818–27. doi: 10.1007/s00066-024-02363-y (PMC12283462; doi:10.1007/s00066-024-02363-y)
Supplement: Supplementary file 4 — Table 4. Fiducial detection rate by BMI group. The BMI groups were devised according to the official BMI classification. Only 48 of 64 patients were analyzed due to missing height and/or weight information in 16 patients (see patients’ characteristics). There was no significant difference between the groups, nor could a trend be detected over the course of the five-fraction treatment. [file 66_2024_2363_MOESM4_ESM.pptx]

## Slide 1
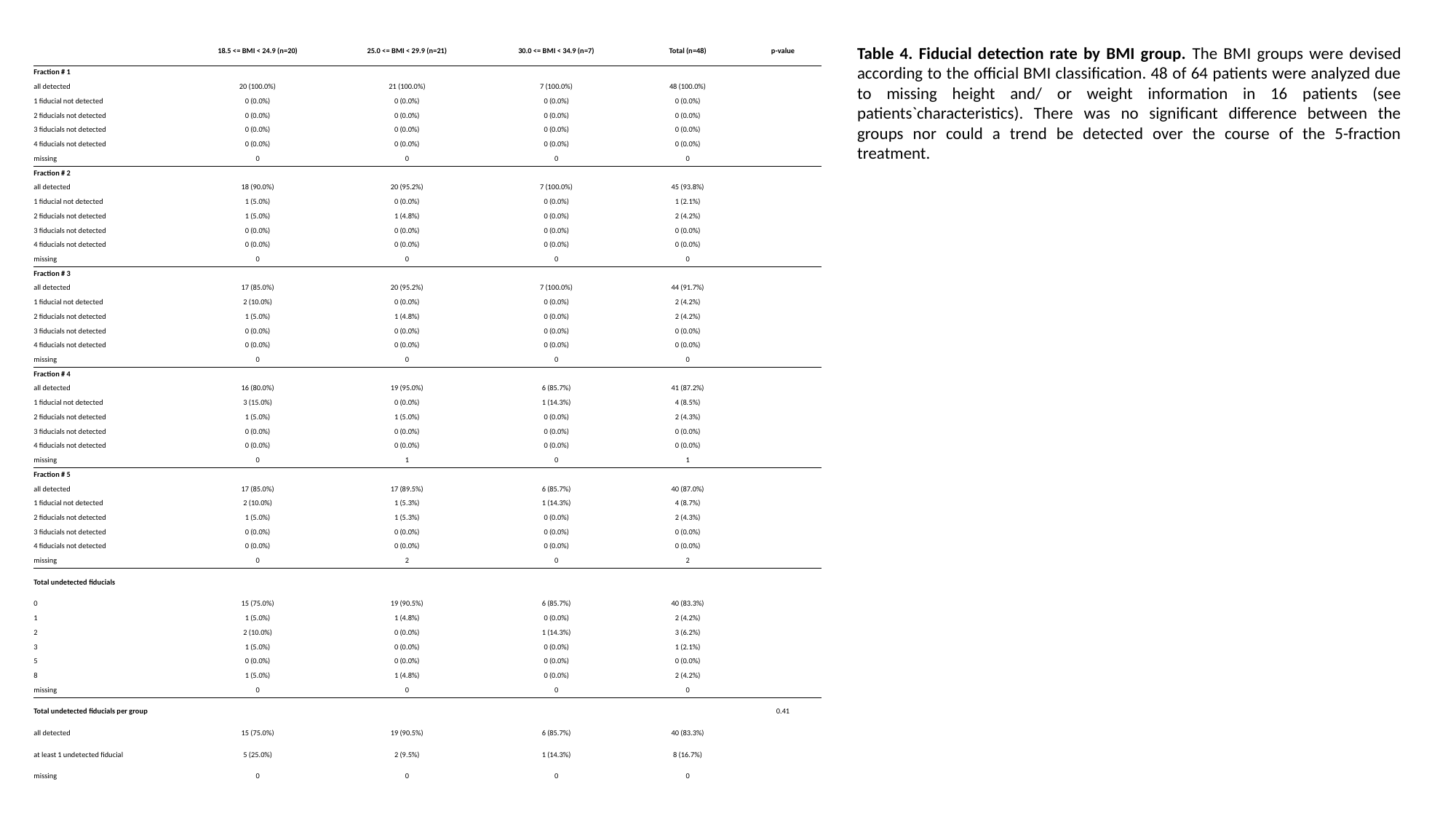

| | 18.5 <= BMI < 24.9 (n=20) | 25.0 <= BMI < 29.9 (n=21) | 30.0 <= BMI < 34.9 (n=7) | Total (n=48) | p-value |
| --- | --- | --- | --- | --- | --- |
| Fraction # 1 | | | | | |
| all detected | 20 (100.0%) | 21 (100.0%) | 7 (100.0%) | 48 (100.0%) | |
| 1 fiducial not detected | 0 (0.0%) | 0 (0.0%) | 0 (0.0%) | 0 (0.0%) | |
| 2 fiducials not detected | 0 (0.0%) | 0 (0.0%) | 0 (0.0%) | 0 (0.0%) | |
| 3 fiducials not detected | 0 (0.0%) | 0 (0.0%) | 0 (0.0%) | 0 (0.0%) | |
| 4 fiducials not detected | 0 (0.0%) | 0 (0.0%) | 0 (0.0%) | 0 (0.0%) | |
| missing | 0 | 0 | 0 | 0 | |
| Fraction # 2 | | | | | |
| all detected | 18 (90.0%) | 20 (95.2%) | 7 (100.0%) | 45 (93.8%) | |
| 1 fiducial not detected | 1 (5.0%) | 0 (0.0%) | 0 (0.0%) | 1 (2.1%) | |
| 2 fiducials not detected | 1 (5.0%) | 1 (4.8%) | 0 (0.0%) | 2 (4.2%) | |
| 3 fiducials not detected | 0 (0.0%) | 0 (0.0%) | 0 (0.0%) | 0 (0.0%) | |
| 4 fiducials not detected | 0 (0.0%) | 0 (0.0%) | 0 (0.0%) | 0 (0.0%) | |
| missing | 0 | 0 | 0 | 0 | |
| Fraction # 3 | | | | | |
| all detected | 17 (85.0%) | 20 (95.2%) | 7 (100.0%) | 44 (91.7%) | |
| 1 fiducial not detected | 2 (10.0%) | 0 (0.0%) | 0 (0.0%) | 2 (4.2%) | |
| 2 fiducials not detected | 1 (5.0%) | 1 (4.8%) | 0 (0.0%) | 2 (4.2%) | |
| 3 fiducials not detected | 0 (0.0%) | 0 (0.0%) | 0 (0.0%) | 0 (0.0%) | |
| 4 fiducials not detected | 0 (0.0%) | 0 (0.0%) | 0 (0.0%) | 0 (0.0%) | |
| missing | 0 | 0 | 0 | 0 | |
| Fraction # 4 | | | | | |
| all detected | 16 (80.0%) | 19 (95.0%) | 6 (85.7%) | 41 (87.2%) | |
| 1 fiducial not detected | 3 (15.0%) | 0 (0.0%) | 1 (14.3%) | 4 (8.5%) | |
| 2 fiducials not detected | 1 (5.0%) | 1 (5.0%) | 0 (0.0%) | 2 (4.3%) | |
| 3 fiducials not detected | 0 (0.0%) | 0 (0.0%) | 0 (0.0%) | 0 (0.0%) | |
| 4 fiducials not detected | 0 (0.0%) | 0 (0.0%) | 0 (0.0%) | 0 (0.0%) | |
| missing | 0 | 1 | 0 | 1 | |
| Fraction # 5 | | | | | |
| all detected | 17 (85.0%) | 17 (89.5%) | 6 (85.7%) | 40 (87.0%) | |
| 1 fiducial not detected | 2 (10.0%) | 1 (5.3%) | 1 (14.3%) | 4 (8.7%) | |
| 2 fiducials not detected | 1 (5.0%) | 1 (5.3%) | 0 (0.0%) | 2 (4.3%) | |
| 3 fiducials not detected | 0 (0.0%) | 0 (0.0%) | 0 (0.0%) | 0 (0.0%) | |
| 4 fiducials not detected | 0 (0.0%) | 0 (0.0%) | 0 (0.0%) | 0 (0.0%) | |
| missing | 0 | 2 | 0 | 2 | |
| Total undetected fiducials | | | | | |
| 0 | 15 (75.0%) | 19 (90.5%) | 6 (85.7%) | 40 (83.3%) | |
| 1 | 1 (5.0%) | 1 (4.8%) | 0 (0.0%) | 2 (4.2%) | |
| 2 | 2 (10.0%) | 0 (0.0%) | 1 (14.3%) | 3 (6.2%) | |
| 3 | 1 (5.0%) | 0 (0.0%) | 0 (0.0%) | 1 (2.1%) | |
| 5 | 0 (0.0%) | 0 (0.0%) | 0 (0.0%) | 0 (0.0%) | |
| 8 | 1 (5.0%) | 1 (4.8%) | 0 (0.0%) | 2 (4.2%) | |
| missing | 0 | 0 | 0 | 0 | |
| Total undetected fiducials per group | | | | | 0.41 |
| all detected | 15 (75.0%) | 19 (90.5%) | 6 (85.7%) | 40 (83.3%) | |
| at least 1 undetected fiducial | 5 (25.0%) | 2 (9.5%) | 1 (14.3%) | 8 (16.7%) | |
| missing | 0 | 0 | 0 | 0 | |
Table 4. Fiducial detection rate by BMI group. The BMI groups were devised according to the official BMI classification. 48 of 64 patients were analyzed due to missing height and/ or weight information in 16 patients (see patients`characteristics). There was no significant difference between the groups nor could a trend be detected over the course of the 5-fraction treatment.

## Slide 2
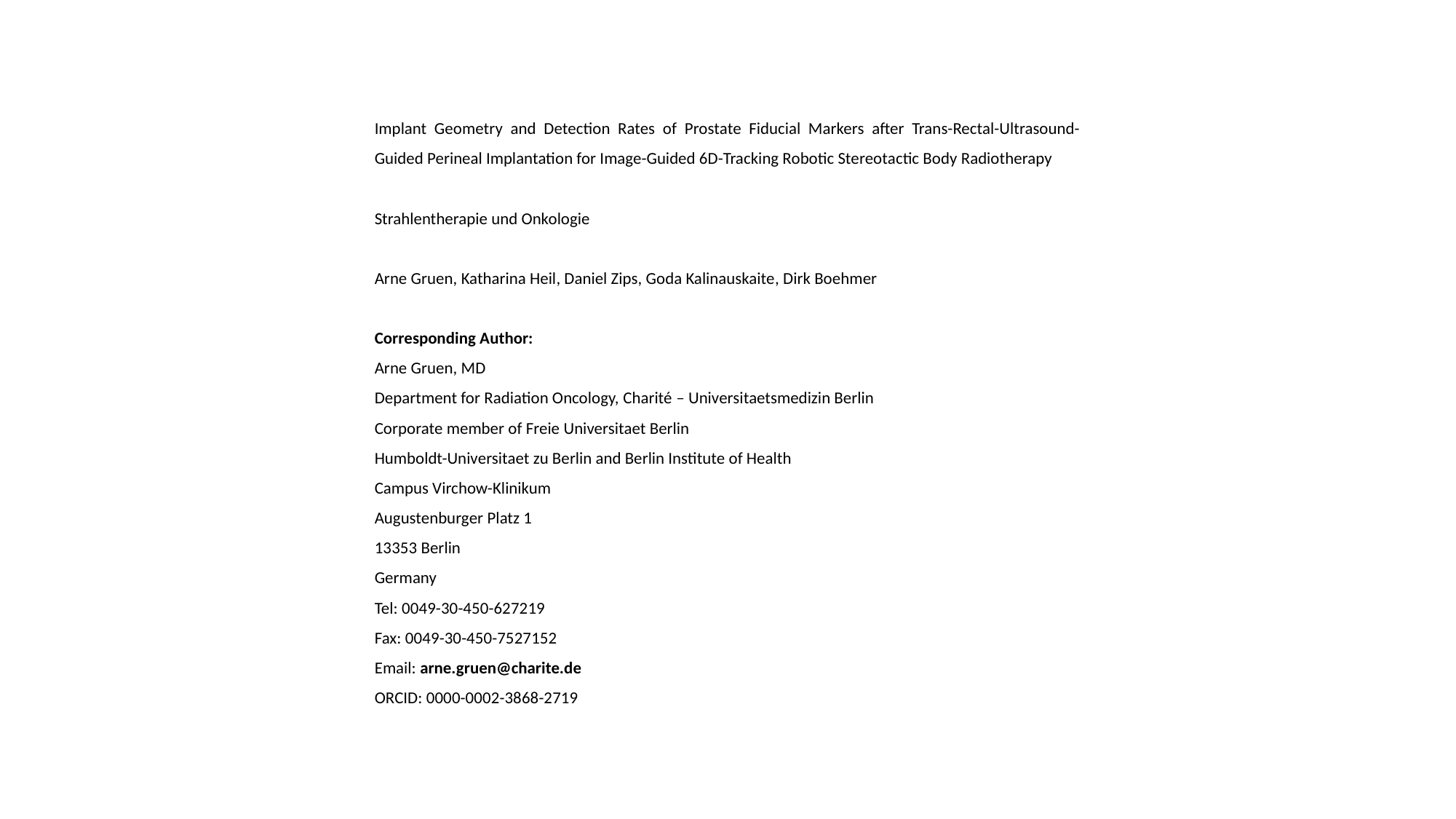

Implant Geometry and Detection Rates of Prostate Fiducial Markers after Trans-Rectal-Ultrasound-Guided Perineal Implantation for Image-Guided 6D-Tracking Robotic Stereotactic Body Radiotherapy
Strahlentherapie und Onkologie
Arne Gruen, Katharina Heil, Daniel Zips, Goda Kalinauskaite, Dirk Boehmer
Corresponding Author:
Arne Gruen, MD
Department for Radiation Oncology, Charité – Universitaetsmedizin Berlin
Corporate member of Freie Universitaet Berlin
Humboldt-Universitaet zu Berlin and Berlin Institute of Health
Campus Virchow-Klinikum
Augustenburger Platz 1
13353 Berlin
Germany
Tel: 0049-30-450-627219
Fax: 0049-30-450-7527152
Email: arne.gruen@charite.de
ORCID: 0000-0002-3868-2719
