# Supplementary material for: Implant geometry and detection rates of prostate fiducial markers after transrectal ultrasound-guided perineal implantation for image-guided 6D-tracking in robotic stereotactic body radiotherapy
Source: Strahlenther Onkol. 2025 Feb 6;201(8):818–27. doi: 10.1007/s00066-024-02363-y (PMC12283462; doi:10.1007/s00066-024-02363-y)
Supplement: Supplementary file 5 — Table 5. Fiducial detection rate by prostate volume group. The prostate volume groups were devised by starting with a normal volume of up to 30 ml and then proceeding in 30 ml steps. N = the whole cohort (64). There was no significant difference between the groups over the course of the five-fraction treatment, although the overall rate of undetected fiducials increased significantly from fraction one to five. [file 66_2024_2363_MOESM5_ESM.pptx]

## Slide 1
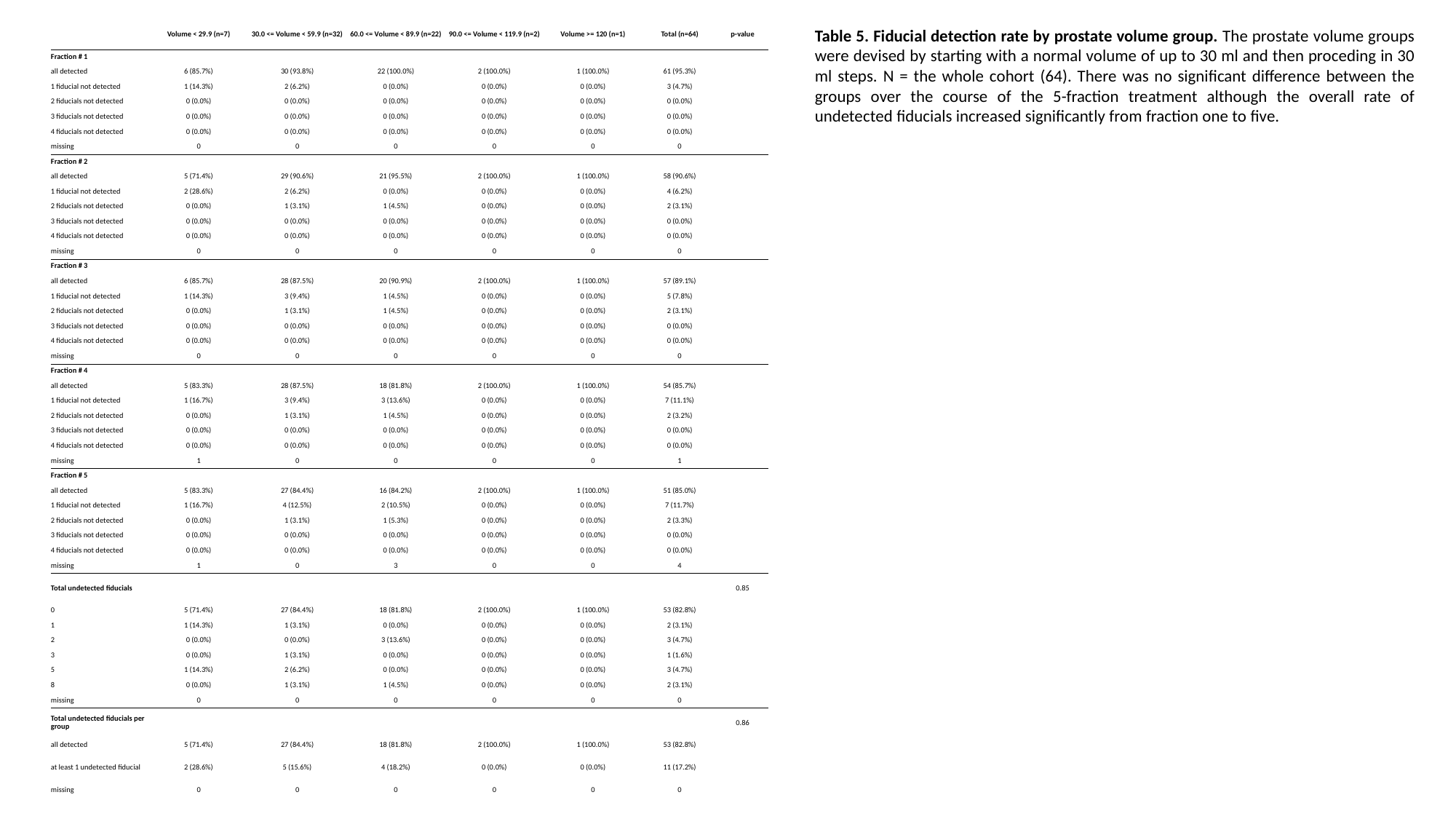

| | Volume < 29.9 (n=7) | 30.0 <= Volume < 59.9 (n=32) | 60.0 <= Volume < 89.9 (n=22) | 90.0 <= Volume < 119.9 (n=2) | Volume >= 120 (n=1) | Total (n=64) | p-value |
| --- | --- | --- | --- | --- | --- | --- | --- |
| Fraction # 1 | | | | | | | |
| all detected | 6 (85.7%) | 30 (93.8%) | 22 (100.0%) | 2 (100.0%) | 1 (100.0%) | 61 (95.3%) | |
| 1 fiducial not detected | 1 (14.3%) | 2 (6.2%) | 0 (0.0%) | 0 (0.0%) | 0 (0.0%) | 3 (4.7%) | |
| 2 fiducials not detected | 0 (0.0%) | 0 (0.0%) | 0 (0.0%) | 0 (0.0%) | 0 (0.0%) | 0 (0.0%) | |
| 3 fiducials not detected | 0 (0.0%) | 0 (0.0%) | 0 (0.0%) | 0 (0.0%) | 0 (0.0%) | 0 (0.0%) | |
| 4 fiducials not detected | 0 (0.0%) | 0 (0.0%) | 0 (0.0%) | 0 (0.0%) | 0 (0.0%) | 0 (0.0%) | |
| missing | 0 | 0 | 0 | 0 | 0 | 0 | |
| Fraction # 2 | | | | | | | |
| all detected | 5 (71.4%) | 29 (90.6%) | 21 (95.5%) | 2 (100.0%) | 1 (100.0%) | 58 (90.6%) | |
| 1 fiducial not detected | 2 (28.6%) | 2 (6.2%) | 0 (0.0%) | 0 (0.0%) | 0 (0.0%) | 4 (6.2%) | |
| 2 fiducials not detected | 0 (0.0%) | 1 (3.1%) | 1 (4.5%) | 0 (0.0%) | 0 (0.0%) | 2 (3.1%) | |
| 3 fiducials not detected | 0 (0.0%) | 0 (0.0%) | 0 (0.0%) | 0 (0.0%) | 0 (0.0%) | 0 (0.0%) | |
| 4 fiducials not detected | 0 (0.0%) | 0 (0.0%) | 0 (0.0%) | 0 (0.0%) | 0 (0.0%) | 0 (0.0%) | |
| missing | 0 | 0 | 0 | 0 | 0 | 0 | |
| Fraction # 3 | | | | | | | |
| all detected | 6 (85.7%) | 28 (87.5%) | 20 (90.9%) | 2 (100.0%) | 1 (100.0%) | 57 (89.1%) | |
| 1 fiducial not detected | 1 (14.3%) | 3 (9.4%) | 1 (4.5%) | 0 (0.0%) | 0 (0.0%) | 5 (7.8%) | |
| 2 fiducials not detected | 0 (0.0%) | 1 (3.1%) | 1 (4.5%) | 0 (0.0%) | 0 (0.0%) | 2 (3.1%) | |
| 3 fiducials not detected | 0 (0.0%) | 0 (0.0%) | 0 (0.0%) | 0 (0.0%) | 0 (0.0%) | 0 (0.0%) | |
| 4 fiducials not detected | 0 (0.0%) | 0 (0.0%) | 0 (0.0%) | 0 (0.0%) | 0 (0.0%) | 0 (0.0%) | |
| missing | 0 | 0 | 0 | 0 | 0 | 0 | |
| Fraction # 4 | | | | | | | |
| all detected | 5 (83.3%) | 28 (87.5%) | 18 (81.8%) | 2 (100.0%) | 1 (100.0%) | 54 (85.7%) | |
| 1 fiducial not detected | 1 (16.7%) | 3 (9.4%) | 3 (13.6%) | 0 (0.0%) | 0 (0.0%) | 7 (11.1%) | |
| 2 fiducials not detected | 0 (0.0%) | 1 (3.1%) | 1 (4.5%) | 0 (0.0%) | 0 (0.0%) | 2 (3.2%) | |
| 3 fiducials not detected | 0 (0.0%) | 0 (0.0%) | 0 (0.0%) | 0 (0.0%) | 0 (0.0%) | 0 (0.0%) | |
| 4 fiducials not detected | 0 (0.0%) | 0 (0.0%) | 0 (0.0%) | 0 (0.0%) | 0 (0.0%) | 0 (0.0%) | |
| missing | 1 | 0 | 0 | 0 | 0 | 1 | |
| Fraction # 5 | | | | | | | |
| all detected | 5 (83.3%) | 27 (84.4%) | 16 (84.2%) | 2 (100.0%) | 1 (100.0%) | 51 (85.0%) | |
| 1 fiducial not detected | 1 (16.7%) | 4 (12.5%) | 2 (10.5%) | 0 (0.0%) | 0 (0.0%) | 7 (11.7%) | |
| 2 fiducials not detected | 0 (0.0%) | 1 (3.1%) | 1 (5.3%) | 0 (0.0%) | 0 (0.0%) | 2 (3.3%) | |
| 3 fiducials not detected | 0 (0.0%) | 0 (0.0%) | 0 (0.0%) | 0 (0.0%) | 0 (0.0%) | 0 (0.0%) | |
| 4 fiducials not detected | 0 (0.0%) | 0 (0.0%) | 0 (0.0%) | 0 (0.0%) | 0 (0.0%) | 0 (0.0%) | |
| missing | 1 | 0 | 3 | 0 | 0 | 4 | |
| Total undetected fiducials | | | | | | | 0.85 |
| 0 | 5 (71.4%) | 27 (84.4%) | 18 (81.8%) | 2 (100.0%) | 1 (100.0%) | 53 (82.8%) | |
| 1 | 1 (14.3%) | 1 (3.1%) | 0 (0.0%) | 0 (0.0%) | 0 (0.0%) | 2 (3.1%) | |
| 2 | 0 (0.0%) | 0 (0.0%) | 3 (13.6%) | 0 (0.0%) | 0 (0.0%) | 3 (4.7%) | |
| 3 | 0 (0.0%) | 1 (3.1%) | 0 (0.0%) | 0 (0.0%) | 0 (0.0%) | 1 (1.6%) | |
| 5 | 1 (14.3%) | 2 (6.2%) | 0 (0.0%) | 0 (0.0%) | 0 (0.0%) | 3 (4.7%) | |
| 8 | 0 (0.0%) | 1 (3.1%) | 1 (4.5%) | 0 (0.0%) | 0 (0.0%) | 2 (3.1%) | |
| missing | 0 | 0 | 0 | 0 | 0 | 0 | |
| Total undetected fiducials per group | | | | | | | 0.86 |
| all detected | 5 (71.4%) | 27 (84.4%) | 18 (81.8%) | 2 (100.0%) | 1 (100.0%) | 53 (82.8%) | |
| at least 1 undetected fiducial | 2 (28.6%) | 5 (15.6%) | 4 (18.2%) | 0 (0.0%) | 0 (0.0%) | 11 (17.2%) | |
| missing | 0 | 0 | 0 | 0 | 0 | 0 | |
Table 5. Fiducial detection rate by prostate volume group. The prostate volume groups were devised by starting with a normal volume of up to 30 ml and then proceding in 30 ml steps. N = the whole cohort (64). There was no significant difference between the groups over the course of the 5-fraction treatment although the overall rate of undetected fiducials increased significantly from fraction one to five.

## Slide 2
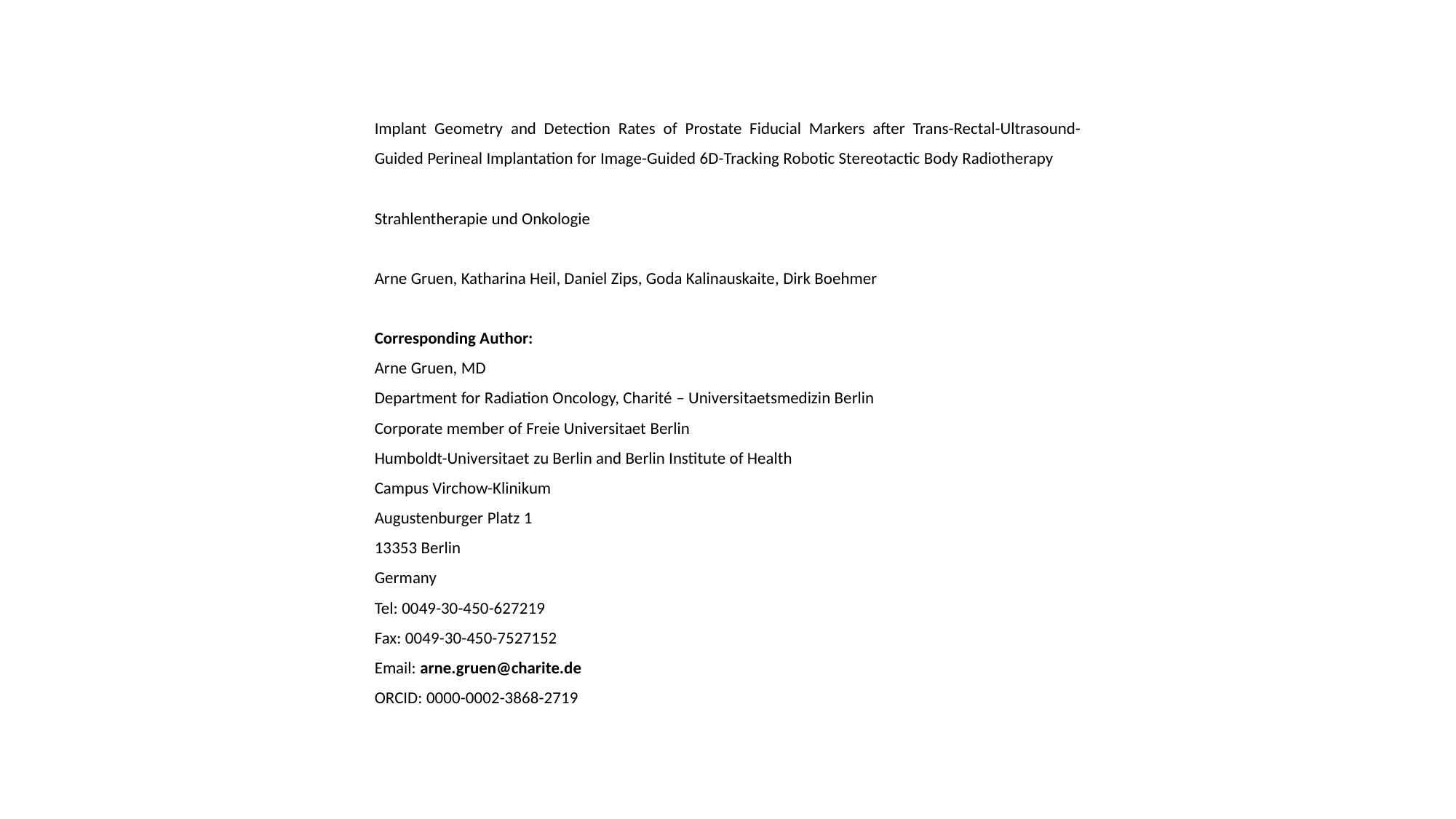

Implant Geometry and Detection Rates of Prostate Fiducial Markers after Trans-Rectal-Ultrasound-Guided Perineal Implantation for Image-Guided 6D-Tracking Robotic Stereotactic Body Radiotherapy
Strahlentherapie und Onkologie
Arne Gruen, Katharina Heil, Daniel Zips, Goda Kalinauskaite, Dirk Boehmer
Corresponding Author:
Arne Gruen, MD
Department for Radiation Oncology, Charité – Universitaetsmedizin Berlin
Corporate member of Freie Universitaet Berlin
Humboldt-Universitaet zu Berlin and Berlin Institute of Health
Campus Virchow-Klinikum
Augustenburger Platz 1
13353 Berlin
Germany
Tel: 0049-30-450-627219
Fax: 0049-30-450-7527152
Email: arne.gruen@charite.de
ORCID: 0000-0002-3868-2719
